# DNA methylation profile is a quantitative measure of biological aging in children

**DOI:** 10.18632/aging.102399

**Published:** 2019-11-22

**Authors:** Xiaohui Wu, Weidan Chen, Fangqin Lin, Qingsheng Huang, Jiayong Zhong, Huan Gao, Yanyan Song, Huiying Liang

**Affiliations:** 1 Institute of Pediatrics, Guangzhou Women and Children’s Medical Center, Guangzhou Medical University, Guangzhou, Guangdong, China; 2 Department of Medical Genetics, School of Basic Medical Sciences, Southern Medical University, Guangzhou, Guangdong, China; 3 Guangdong Technology and Engineering Research Center for Molecular Diagnostics of Human Genetic Diseases, Guangzhou, Guangdong, China; 4 Guangdong Province Key Laboratory of Psychiatric Disorders, Guangzhou, Guangdong, China; 5 Department of Cardiovascular Surgery, Guangzhou Women and Children’s Medical Center, Guangzhou Medical University, Guangzhou, Guangdong, China; 6 The Guangdong Early Childhood Development Applied Engineering and Technology Research Center, Guangzhou Women and Children’s Medical Center, Guangzhou Medical University, Guangdong, China

**Keywords:** children, biological age, prediction, methylation, age-related diseases

## Abstract

DNA methylation changes within the genome can be used to predict human age. However, the existing biological age prediction models based on DNA methylation are predominantly adult-oriented. We established a methylation-based age prediction model for children (9-212 months old) using data from 716 blood samples in 11 DNA methylation datasets. Our elastic net model includes 111 CpG sites, mostly in genes associated with development and aging. The model performed well and exhibited high precision, yielding a 98% correlation between the DNA methylation age and the chronological age, with an error of only 6.7 months. When we used the model to assess age acceleration in children based on their methylation data, we observed the following: first, the aging rate appears to be fastest in mid-childhood, and this acceleration is more pronounced in autistic children; second, lead exposure early in life increases the aging rate in boys, but not in girls; third, short-term recombinant human growth hormone treatment has little effect on the aging rate of children. Our child-specific methylation-based age prediction model can effectively detect epigenetic changes and health imbalances early in life. This may thus be a useful model for future studies of epigenetic interventions for age-related diseases.

## INTRODUCTION

Due to the declining fertility rate and the increasing life expectancy, the world is on the brink of a demographic milestone: adults above the age of 65 will soon outnumber children under the age of 5 [1–3]. Population aging is accompanied by increases in illness, disability and dependency [4]. Consequently, noncommunicable diseases that more commonly occur in adults and older people are imposing the greatest burden on global health. Extending the period of life free of disability and disease is the key to limiting health and social costs. The most promising approach to this end is to identify age-related biological changes in body function or structure that are more accurate than the chronological age in predicting the future onset of age-related diseases or the remaining years of life [5].

Biomarkers of biological aging can be classified as molecular markers (based on DNA, RNA, etc.) or phenotypic biomarkers (based on anthropometric data such as bone age assessment, blood pressure, lipid levels, etc.) [5]. However, most studies of such biomarkers have been conducted in animals or older individuals [6–9]. Animals with short lifespans are typically not accurate models of the complex multifactorial exposures during human aging. Furthermore, most elderly study participants already suffer from age-related diseases. The theory of the fetal origins of adult disorders [10–13] proposes that many health problems in adults or the elderly are rooted in early life experiences and living conditions. Thus, interventions to reverse or delay age-related diseases and aging itself must be performed in childhood. The lack of tools to quantify aging in children is a significant obstacle to this goal.

Thus far, the most remarkable biological age predictor has been the epigenetic clock. Hannum et al. built a quantitative model of aging by measuring over 450,000 CpG markers in whole blood samples from 656 human subjects aged 19 to 101 years [14]. Horvath et al. developed a biomarker of aging called the multi-tissue predictor based on DNA methylation levels [15]. Using only three CpG sites, Weidner et al. constructed an age prediction model that was more precise than techniques based on telomere length [16]. The above studies demonstrated the feasibility of biological age prediction based on DNA methylation, but these models were mostly focused on adults. Although some of these predictive models included samples from children, the large age range (0–101 years old) and age unit (years) of these models reduced their accuracy both in predicting the biological ages of children (0 - 18 years old) and in revealing biologically relevant epigenetic abnormalities.

There has been some progress in DNA methylation research in children, but there are still many problems to be solved. For instance, Alisch et al. found 2078 age-associated CpG sites in boys (3–17 years old), but did not propose operational quantitative tools [17]. Almstrup et al. only used the data from 51 healthy children (5–16 years old) before and after pubertal onset to predict adolescent development [18]. Freire-Aradas et al. built a preliminary age prediction model using a dataset of 180 donors (2–18 years old) with the EpiTYPER^®^ DNA methylation analysis system [19]. None of these studies completely covered the age range from 0 to 18 years, and each study used a single sample data type with a small number of samples, so the results were not highly accurate or applicable.

To delineate the aging pattern precisely throughout childhood, we analyzed DNA methylation datasets from a large cohort of children to construct a child-specific methylation-based age prediction model covering the whole age period from 0 to 18 years with a small age unit (months). Our model is a new tool for quantifying health imbalances and monitoring predictors of age-related diseases early in life, and thus may facilitate early prevention and intervention.

## RESULTS

### Establishment of a child-specific methylation-based age prediction model

### Characteristics of the DNA methylation datasets

**Figure 1:**
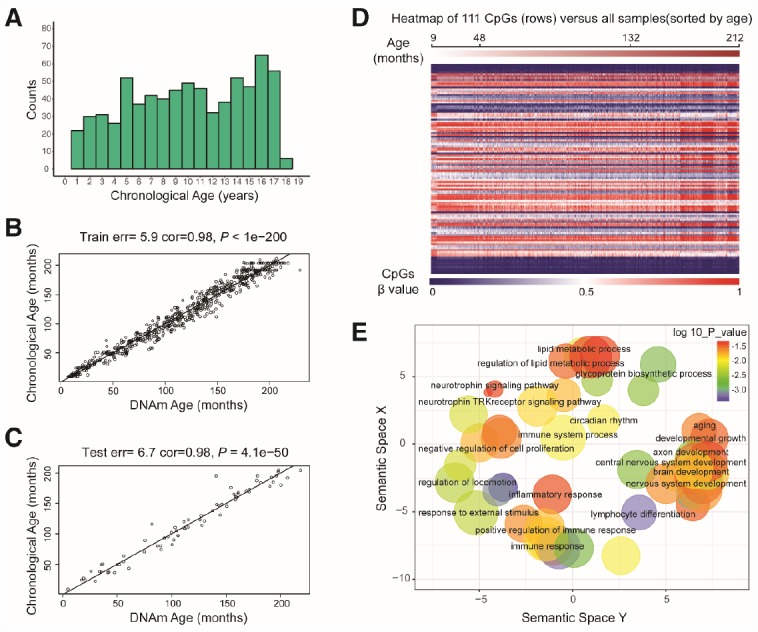
**Characteristics of the prediction model.** (**A**) Histogram of the age distribution for healthy children. The *x*-axis represents the chronological age of the individuals (age unit is years) and the *y*-axis (counts) represents the number of individuals. (**B**) Scatterplot of the DNA methylation (DNAm) age (*x*-axis) against the chronological age (*y*-axis) for the individuals in the training sets (age unit is months). For the training data, the correlation between the DNAm age and chronological age was 0.98, and the error (median absolute difference) was 5.9 months. (**C**) Scatterplot of the DNAm age (*x*-axis) against the chronological age (*y*-axis) for individuals in the test sets (age unit is months). For the test data, the correlation was 0.98 and the error was 6.7 months. (**D**) Heatmap of the DNA methylation levels of 111 CpG sites. Each row represents one CpG site, and the blue to red color spectrum represents β values from 0 to 1. The individuals are sorted by age (9 to 212 months), and it can be seen that the DNA methylation levels change with age. (**E**) Gene ontology analysis of the 111 CpG sites revealed several ontologies (*P* < 0.05) that may be associated with development and aging. Biological process gene ontologies were plotted in a sematic space with REVIGO, which groups related ontologies together.

We obtained publicly available DNA methylation datasets that were generated with the Illumina 27K or Illumina 450K array platform. Data from 716 healthy children aged 9 to 212 months from 11 different datasets were used to build the quantitative model (Table 1, Figure 1A). These healthy children included 529 boys and 187 girls (Supplementary Figure 1A). Nearly half of the samples (46.5%) were assessed on the Illumina 450K platform (Supplementary Figure 1B). We only studied the 21,979 CpG sites that were present on both Illumina platforms. For simplicity and accuracy, we discarded markers on sex chromosomes and markers with more than 10 missing values across the datasets. DNA methylation levels were recorded as β values between 0 (completely unmethylated) and 1 (completely methylated). To study the link between disease and methylation age in childhood, we also analyzed the datasets of children with diseases (Supplementary Table 1). Details on the above datasets and the data preprocessing steps are provided in the Materials and Methods.

**Table 1:** Summary details of the DNA methylation datasets from children.

**ID**	**Availability**	**Methylation array**	***n***	**Age(months)**	**Gender**	**Ethnicity**	**Citation**
1	GSE27097	Illumina 27K	334	43.0-212.0	M: 334	white: 266, asian: 14, african-amer: 3, other: 9, more-than-one-race: 35, native-american: 1, native-hawaiian: 1, not-specified: 5	Alisch et al.
2	GSE32148	Illumina 450K	13	60.0-210.0	M: 1 F: 8	null	Harris et al.
3	GSE57484	Illumina 27K	9	120.24-127.8	M: 9	null	Voisin et al.
4	GSE64495	Illumina 450K	18	27.6-129.6	M: 6 F: 12	null	Brunet et al.
5	E-MTAB-4187	Illumina 450K	84	67.0-196.7	M: 51 F: 33	null	Almstrup et al.
6	GSE34257	Illumina 27K	15	9.0	M: 7 F: 8	null	Khulan et al.
7	GSE36054	Illumina 450K	127	12.0-203.0	M: 76 F: 51	Black: 69, White: 8, Other: 42, Asian: 3, Unknown: 5	Alisch et al.
8	GSE23638	Illumina 27K	18	31.1-204.0	M: 9, F: 9	null	Chen et al.
9	GSE41037	Illumina 27K	7	180.0-207.3	M: 6 F: 1	null	Horvath et al.
10	GSE52588	Illumina 450K	3	120.0-180.0	M: 1 F: 2	null	Bacalini et al.
11	GSE73103	Illumina 450K	88	158.2-204	M: 25 F: 63	null	Voisin et al.

### A precise DNA methylation age prediction model in children

**Figure 2:**
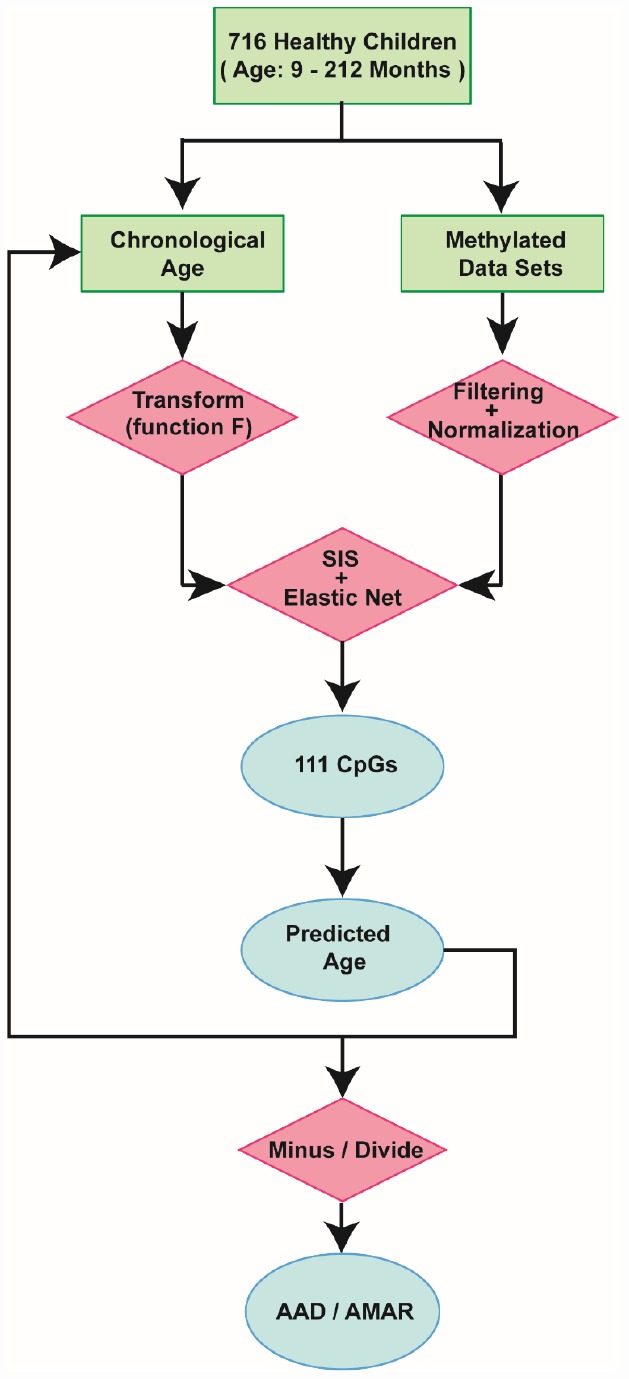
**Schematic of the prediction model.** A flow diagram of the child-specific methylation-based age prediction model. The green boxes represent the input data, the red diamonds represent the analysis methods and the blue ovals represent the prediction results. AAD: age acceleration difference; AMAR: apparent methylation aging rate; SIS: sure independence screening.

By combining sure independence screening [20] and penalized multivariate (elastic net) regression [21], we established a child-specific methylation-based age prediction model in the training cohort, and called the prediction value the DNA methylation age (Figure 2). K-fold cross-validation (k = 10) [22] was implemented to divide the training sets and test sets. The optimal model included 111 CpG sites that were accurately predictive of age (regression coefficients in Supplementary Table 2). In the training sets, the model was highly accurate, with a 98% correlation between age and predicted age, and an error of 5.9 months (Figure 1B). The accuracy remained the same when this model was validated on the test sets, as there was a 98% correlation between age and predicted age, and the error was 6.7 months (Figure 1C). The β values of the 111 CpG sites exhibited some certain trends with increasing age, although most of these changes were not very dramatic. The effects of age on the 111 CpG sites were visualized on a heat map, which showed the trends in DNA methylation across subjects (Figure 1D).

### Age-related CpG sites associated with development and aging

To determine the biological functions of the 111 age-related CpG sites, we searched for significantly enriched GO terms (biological processes, cellular components and molecular functions, *P* < 0.05) and KEGG signaling pathways among the genes associated with these CpG sites. The top 20 GO terms and KEGG pathways are listed in Supplementary Figure 1. The GO terms were then drawn in a semantic space, and similar terms were combined. The results revealed clusters associated with developmental growth, immune responses, metabolic regulation and age-related diseases such as systemic lupus erythematosus, rheumatoid arthritis and cancer (Figure 1E, Supplementary Figure 1C and 1D, Supplementary Table 3).

### Comparing the child-specific age predictor with other age predictors

**Figure 3:**
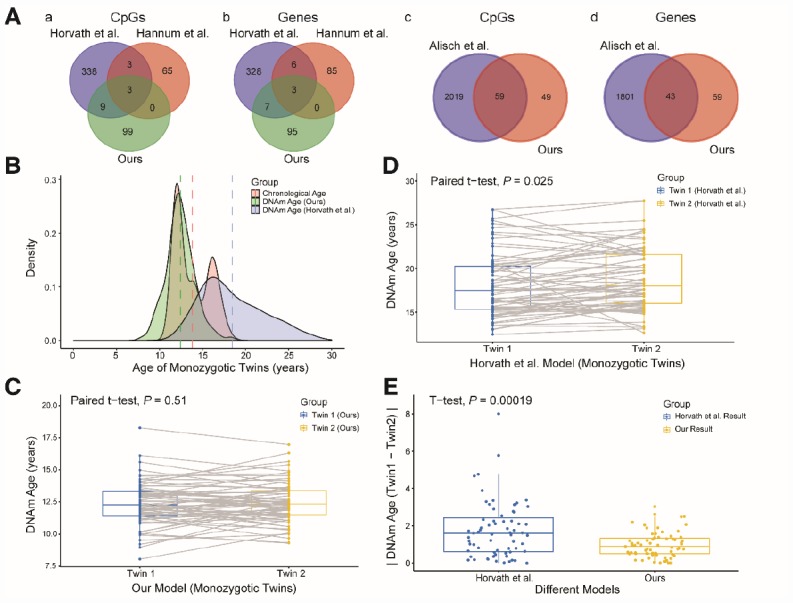
**Comparison and verification of our model.** (**A**) (a and b) Venn diagrams of the CpG sites (a) and genes associated with the CpG sites (b) selected from the three models. (c and d) Venn diagrams of the CpG sites (c) and genes (d) associated with age from our study and the study of Alisch et al. (**B**) Density plot of age and DNA methylation (DNAm) age. The red peak represents the chronological age, the green peak represents the DNAm age predicted by our model, and the blue peak represents the DNAm age predicted by Horvath et al. Dashed lines represent mean values. (**C**) Boxplot comparing the DNAm ages predicted by our model for monozygotic twins (paired t-test, n = 67 each for twins 1 and 2). The blue box indicates twin 1 and the yellow box indicates twin 2. (**D**) Boxplot comparing the DNAm ages predicted by the model of Horvath et al. for monozygotic twins (paired t-test, n = 67 each for twins 1 and 2). The blue box indicates twin 1 and the yellow box indicates twin 2. (**E**) Boxplot comparing the absolute values of the DNAm age differences of monozygotic twins predicted by the two models (two-sided t-test, n = 67). The blue box indicates the results from Horvath et al. and the yellow box indicates our results.

We then explored the CpG sites selected for different age prediction models. There were only three overlapping sites among Hannum’s 71 CpGs [14], Horvath’s 353 CpGs [15] and our 111 CpGs (Figure 3A a, b): cg04474832, cg09809672 and cg19722847. These sites are associated with the *ABHD14A*, *EDARADD* and *IPO8* genes, respectively. Of the remaining 108 sites in our study, 59 (54.6%) overlapped with 2,078 previously-identified age-related sites in children [17]; these sites involved 43 genes (Figure 3A c, d). We also compared our model with the model established by Freire-Aradas et al. [19], and obtained only two overlapping genes: *EDARADD* and *PRKG2*.

Since different data and methods were used to construct the above models, it was not possible to identify the most accurate model simply by comparing the error values of the models for the test sets. Thus, to further explore the accuracy and applicability of our model, we validated it with data from 67 pairs of monozygotic twins in the dataset GSE56105 [23]. We took this approach because the DNA methylation ages of healthy monozygotic twins who share the same genetic background and living environment should theoretically be similar. First, we used our model and the commonly used multi-tissue predictor [15] to calculate the DNA methylation ages of the monozygotic twins. The predictions from our model were closer to the actual ages of the twins, and the distribution of DNA methylation ages was more concentrated than that of the multi-tissue predictor (Figure 3B). Second, we compared the DNA methylation ages of twins 1 and 2 calculated by these two models. The predicted DNA methylation ages of twins 1 and 2 did not differ significantly when our model was used (Figure 3C, *P* = 0.51, paired t-test), while they did differ significantly when the multi-tissue predictor was used (Figure 3D, *P* = 0.025, paired t-test). Finally, we compared the absolute values of the DNA methylation age differences between twins 1 and 2 calculated by the two models. The absolute values of the two models differed significantly (Figure 3E, *P* < 0.01, t-test), and the values calculated by our model were closer to zero than those calculated by the multi-tissue predictor. Therefore, our prediction model performed better than the multi-tissue predictor in estimating children’s DNA methylation ages based on blood samples.

We could not compare the accuracy of our model with that of the model established by Freire-Aradas et al. [19] because the authors did not report their predicted age calculation formula and data; however, the error of 1.25 years (15 months) reported by Freire-Aradas et al. [19] was larger than the error of our model (6.7 months).

### Aging patterns in children revealed by our model

Our aging model not only predicted the age of most children with high accuracy, but also revealed individual biological differences and aging trends in the pediatric population [14, 15]. To examine whether these differences were true biological differences (rather than measurement error or intrinsic variability), we used our aging model for two measurements of age acceleration. The first, called the age acceleration difference (AAD), is the DNA methylation age minus the chronological age. The second, called the apparent methylation aging rate (AMAR), is the DNA methylation age divided by the chronological age.

### Age acceleration in children seems not to be influenced by gender or ethnicity

We then explored the association of the AAD and AMAR with the potentially clinically relevant factors of gender and ethnicity. In terms of gender, the mean AAD and AMAR values in all the healthy children’s samples were -0.01 months and 1.01, respectively. The AAD and AMAR values for boys were 0.003 months and 1.006, respectively, while the values for girls were -0.040 months and 1.020, respectively. Neither the AAD nor the AMAR differed significantly between boys and girls (AAD: *P* = 0.92, Wilcoxon test; Supplementary Figure 2A), although the AMAR was approximately 1.4% faster in girls than in boys (Supplementary Figure 2B). In contrast, in adults, the AMAR was reported to be 4% faster in men than in women [14]. This difference may be due to the fact that girls develop earlier than boys [24–26]. Regarding ethnicity, neither the AAD nor the AMAR differed significantly among children of different ethnicities (AAD: *P* = 0.9, AMAR: *P* = 0.26, ANOVA; Supplementary Figure 2C and 2D).

### Age acceleration is the greatest in mid-childhood

**Figure 4:**
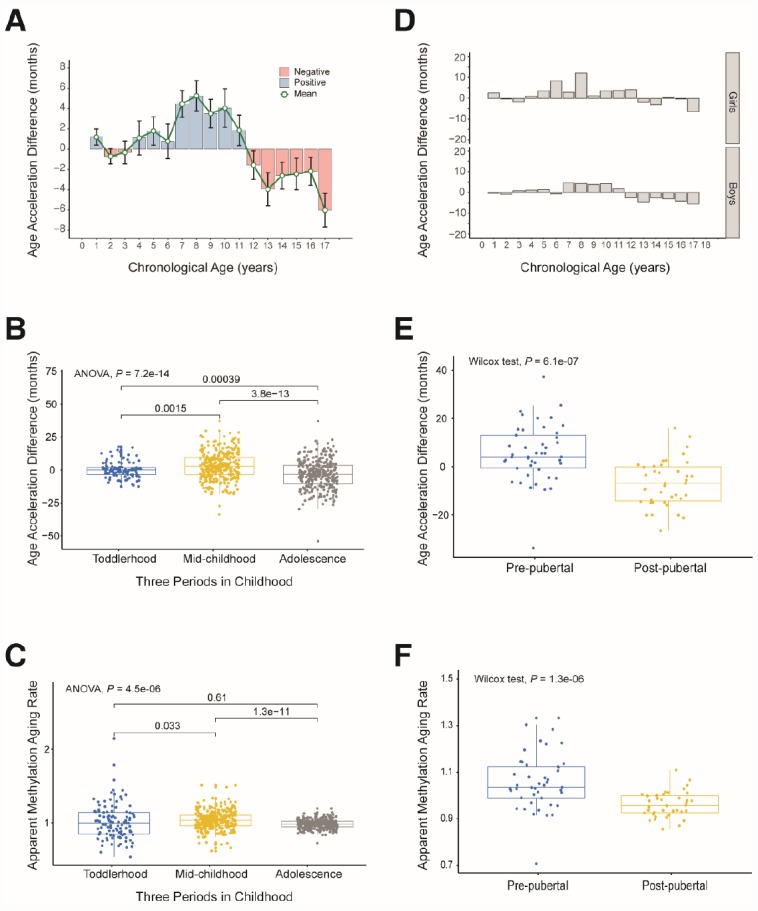
**Age acceleration in different periods of childhood.** (**A**) Histogram of the mean value distribution of the age acceleration difference for all individuals. A pink column indicates a negative value, meaning that the average difference between the DNA methylation age and the chronological age is less than zero. A blue column indicates a positive value, meaning that the average difference between the DNA methylation age and the chronological age is greater than zero. The green circle represents the average difference between the DNA methylation age and the chronological age (age unit is years). (**B**) Boxplot of the age acceleration difference during different periods of childhood. The blue box indicates toddlerhood, the yellow box indicates mid-childhood and the gray box indicates adolescence. (**C**) Boxplot of the apparent methylation aging rate during different periods of childhood. The box colors have the same meaning as above. (**D**) Histograms of the mean value distribution of the age acceleration difference for girls and boys, respectively. (**E**) Boxplot comparing the age acceleration difference between pre-pubertal and post-pubertal individuals. The blue box indicates pre-pubertal individuals and the yellow box indicates post-pubertal individuals. (**F**) Boxplot comparing the apparent methylation aging rate between pre-pubertal and post-pubertal individuals. The box colors are the same as above.

We observed a trend in age acceleration in healthy children between the ages of 0 and 18 years. The age acceleration was close to zero before the age of 4, gradually rose after the age of 5, and fell to a negative value after the age of 12 (Figure 4A). To further explore the aging pattern in children, we divided childhood into three periods: toddlerhood (0–4 years), mid-childhood (5–11 years) and adolescence (12–18 years). We found that the AAD and AMAR were significantly greater in mid-childhood than in toddlerhood, and were significantly lower in adolescence than in mid-childhood (AAD: *P* = 7.2×10^-14^, AMAR: *P* = 4.5×10^-6^, ANOVA; Figure 4B and C). The same phenomenon was observed after sex stratification. Moreover, in mid-childhood, the aging rate seemed to be marginally faster in girls than in boys (Figure 4D, Supplementary Figure 3). These differences in the AAD and AMAR at different stages of childhood indicate that the aging rate of children is not completely consistent with the growth curve, which may be related to the development of several major organ systems.

To verify the above results, we analyzed an independent dataset from the study of Almstrup et al. [18]. These authors described longitudinal whole-genome changes in DNA methylation in peripheral blood samples (n = 84) before and after adolescence in 42 healthy children. Coincidentally, the pre-puberty (5.6–11.3 years) and post-puberty (12.2–16.4 years) age segments in their study were almost the same as our mid-childhood and adolescent age settings, so we could use this dataset to verify our results. As expected, the rate of aging was significantly higher before puberty than after puberty (AAD: *P* = 6.1×10^-7^, AMAR: *P* = 1.3×10^-6^, Wilcoxon test; Figure 4E and 4F). The conclusion of the study by Almstrup et al. also confirmed this finding.

### Association of DNA methylation age with disease

To investigate the association of the DNA methylation age with potential health problems in children, we analyzed three datasets, which respectively focused on diseases, short-term interventions and long-term environmental exposures in children.

### Age acceleration and autism

Using the dataset GSE27044 (details in Supplementary Table 4), we analyzed the association of the three types of autism (autism, autism spectrum disorder and Asperger syndrome) with the DNA methylation age (Supplementary Figure 4A) [17]. We found no significant difference in the AAD or AMAR between these three types of autistic children and their unaffected siblings (AAD: *P* = 0.47, AMAR: *P* = 0.098, ANOVA; Supplementary Figure 4B and 4C).

**Figure 5:**
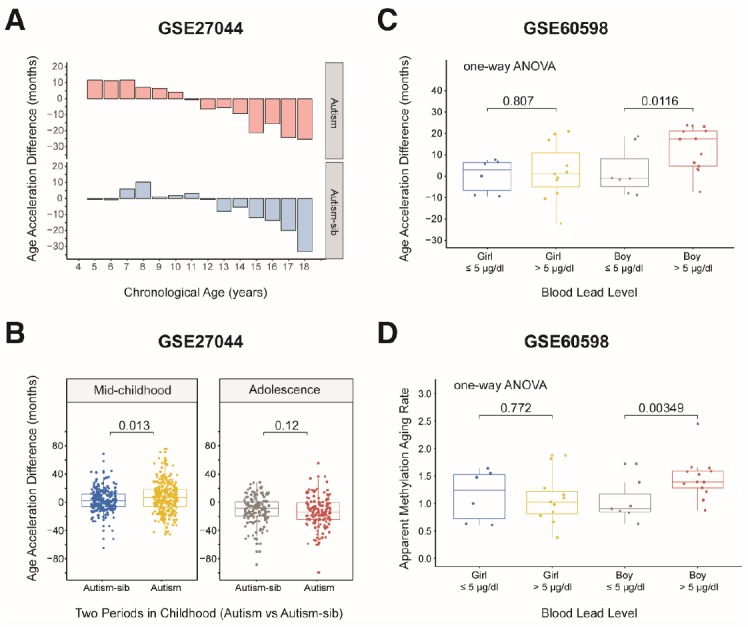
**Age acceleration in children with diseases.** (**A**) Histograms of the mean value distribution of the age acceleration difference in autistic children and their unaffected siblings. The pink columns indicate the children with autism, while the blue columns indicate their unaffected siblings (‘Autism-sib’). (**B**) Boxplot comparing the age acceleration difference between autistic children and their unaffected siblings during two periods of childhood. The blue box and the yellow box indicate the autistic children and their unaffected siblings, respectively, in mid-childhood. The gray box and the red box indicate the autistic children and their unaffected siblings, respectively, in adolescence. (**C**) Boxplot comparing the age acceleration differences of boys and girls with different blood lead levels. A cutoff value of 5 μg/dL was used for the blood lead level. The jitter points represent the age acceleration differences of individual samples. (**D**) Boxplot comparing the apparent methylation aging rates of boys and girls with different blood lead levels. A cutoff value of 5 μg/dL was used for the blood lead level. The jitter points represent the apparent methylation aging rates of individual samples.

We then assessed whether children with the first type of autism conformed to the aforementioned pattern in which the aging rate was significantly greater in mid-childhood. We grouped the samples according to the previous criteria, but there was no toddler group because there was only one child aged less than 48 months. As expected, the rate of aging was significantly higher in mid-childhood than in adolescence in the autistic children (AAD: *P* = 2.2×10^-16^, Wilcoxon test; Supplementary Figure 4D). Surprisingly, the aging rate was significantly higher in autistic children than in their unaffected siblings in mid-childhood (AAD: *P* = 0.013, Wilcoxon test; Table 2, Figure 5A and 5B), but this difference was not observed in adolescence. This finding suggests that autistic children age faster than healthy children in mid-childhood. The above results also indicate that it is worthwhile to examine the significance of mid-childhood and to analyze subgroups throughout childhood (0–18 years).

**Table 2:** Overview of two measures of age accelerations evaluating the effect of autism.

**Group**	**Sample (n)**	Age (months)^*^	**Mean±SD**	**△Mean**	**95%CI**	**Cohen’s d**	**95%CI**	**P value**	**Power (%)**
**AAD: DNAm age – Age (months)**									
Case^†^	260	48 - 132	6.79±20.67	3.96±1.66	[0.71, 7.21]	0.215	[0.036, 0.394]	0.013	77.0
Control^†^	226	48 - 132	2.83±15.83						
**AMAR: DNAm age / Age**									
Case^†^	260	48 - 132	1.09±0.25	0.1±0.02	[0.06, 0.14]	0.263	[0.084, 0.442]	0.003	91.5
Control^†^	226	48 - 132	1.03±0.20						

* min – max.

† Case: Autism; Control: Unaffected siblings.

### Age acceleration and short-term rhGH treatment

We analyzed data from 48 peripheral blood samples taken from 24 patients prior to the first dose and after four days of rhGH treatment (GSE57205 [27]). The rate of aging in these patients did not differ significantly before and after rhGH treatment (*P* > 0.05, t-test; Supplementary Figure 4E). The diagnoses leading to rhGH treatment in this cohort were classical GH deficiency (classical GH deficiency [STH-D], n = 7; panhypopituitarism [PAN], n = 1; and small for gestational age [SGA], n = 1), neurosecretory dysfunction leading to GH deficiency (NSD, n = 6), SGA with a lack of catch-up growth (SGA, n = 7), qualitative GH deficiency (Q-STH-D, n = 2, Kowarski syndrome), Turner syndrome (n = 1) and primary insulin-like growth factor 1 deficiency (n = 1). We compared the AAD values of patients with the first three diagnoses before the treatment; the remaining types had too few samples and were not included in the analysis. The AAD was lower in the NSD group than in the other two groups (*P* < 0.05, t-test; Supplementary Figure 4F), which may reflect the underlying neurosecretory dysfunction in NSD patients [28, 29]. These results suggest that short-term rhGH treatment does not significantly influence age acceleration, although different types of GH deficiency may be associated with different rates of age acceleration.

### Age acceleration and lead exposure early in life

We then evaluated a dataset of 42 dry blood spots from 24 boys and 18 girls aged three months to five years who were exposed to lead in the environment (from GES60598 [30]). The grouping criterion was a blood lead level (BLL) > 5 μg/dL, the maximum safe limit recommended by the Advisory Committee on Childhood Lead Poisoning Prevention in 2012. Boys are more sensitive than girls to lead exposure [31, 32]; thus, we expected to observe significantly greater changes in the AAD and AMAR in boys than in girls if the DNA methylation age was associated with lead exposure. When we grouped the samples by gender, we found that this indeed was the case. The AMAR differed significantly between boys with BLL values > 5 μg/dL and ≤ 5 μg/dL in a t-test (*P* = 0.033, Table 3), but this phenomenon was not observed in girls. To control for spurious findings, we performed one-way ANOVA to assess the association between lead exposure and DNA methylation age, controlling for the age of the children and the age, gestational age and smoking status of their mothers. The AAD and AMAR were significantly higher in lead-exposed boys (BLL > 5 μg/dL) than in nonexposed boys (BLL ≤ 5 μg/dL) (AAD: *P* = 0.0116, AMAR: *P* = 0.00349; Figure 5C and 5D).

**Table 3:** Overview of two measures of age accelerations evaluating the effect of lead exposure early.

**Group**	**Sample (n)**	Age (months)^*^	**Mean±SD**	**△Mean**	**95%CI**	**Cohen’s d**	**95%CI**	**P value**	**Power (%)**
**AAD: DNAm age – Age (months)**									
BLL^†^ > 5 μg/dl	13	12 - 60	12.74±9.97	10.67±5.05	[0.77, 20.57]	1.027	[0.055, 1.999]	0.058	63.8
BLL^†^ ≤ 5 μg/dl	7	9 - 48	2.07±11.18						
**AMAR: DNAm age / Age**									
BLL^†^ > 5 μg/dl	13	12 - 60	1.46±0.37	0.42±0.17	[0.09, 0.75]	1.135	[0.151, 2.119]	0.033	76.2
BLL^†^ ≤ 5 μg/dl	7	9 - 48	1.04±0.37						

* min – max.

† BLL: Blood lead level.

To sum up, DNA methylation age abnormalities may be associated with certain health problems in children. Our child-specific methylation-based age prediction model can be used to reveal aging trends, to study the relationship of age acceleration with diseases and environmental factors impacting children’s growth and development, and to explore the influence of these factors on children’s health and longevity.

## DISCUSSION

Recently, several age prediction models based on DNA methylation have been published. However, due to their large age range (0–101 years old) and age unit (years), most of these models cannot accurately predict the biological ages of children [14–16]. Although a few studies have examined DNA methylation in childhood, they used small amounts of data detected on infrequently used platforms and provided no explicit quantitative tools [17–19]. Therefore, we employed data from two commonly used methylation chips (Illumina 27K and Illumina 450K array platforms) to construct a child-specific methylation-based age prediction model covering the entire period of childhood (0–18 years old) with a small age unit (months). Our model has the following advantages over other age prediction models: a) it comprehensively reflects the aging patterns in childhood; b) it uses months as the age unit, thus increasing the accuracy of the prediction results; c) it solves the problem of insufficient variable selection methods by using sure independence screening [20] before multiple linear regression (elastic net) [21], as the former performs better when the dimension of the predictor p is much larger than the sample size n; and d) it is based on data from whole blood samples analyzed with two types of chips (Illumina 27K array and Illumina 450K arrays), and thus can enhance the practical diagnostic design and analysis of samples collected from other studies. Although the multi-tissue age predictor has broader applicability than our model, tissue specificity can influence the accuracy of predictions. We will later validate and optimize our model in other tissue types. Moreover, we will examine how to better balance the accuracy and applicability of the model.

There were only three overlapping sites between the adult-directed age prediction model and our child-specific methylation-based age prediction model. The cg09809672 site is associated with the EDAR-associated death domain gene (*EDARADD*), which has been linked to ectodermal dysplasia, especially hypohidrotic ectodermal dysplasia [16, 33–35]. The cg04474832 and cg19722847 sites are associated with the *ABHD14A* and IPO8 genes, respectively. *ABHD14A* may be involved in the development of granule neurons, while IPO8 is involved in a common bone marrow mesenchymal stem cell degenerative joint disease [36]. These three sites are most likely associated with aging throughout the entire life process, but the low number of overlapping sites indicates that children have specific age-related methylation characteristics.

Surprisingly, 54.6% of the remaining 108 sites screened by our model overlapped with previously determined age-related sites in children [17]. This high overlap rate indicates that our model effectively reflects the specific age-related methylation changes in children. Alisch et al. [17] reported that age-related DNA methylation changes in peripheral blood occurred more rapidly during childhood; thus, our choice of peripheral blood samples was appropriate. We identified the gene loci of these 59 CpG sites and annotated them using GO terms. The genes were involved in a concentrated set of developmental processes and immune functions, consistent with the known associations between DNA methylation changes and age-related immune system activities [37, 38].

When we compared our model with that established by Freire-Aradas et al. [19], we identified two overlapping genes. *EDARADD* was introduced in the previous paragraph, and has appeared in most of the relevant studies [15, 16]. The protein encoded by the *PRKG2* gene regulates intestinal fluid balance, and changes in its methylation level correlate highly with age in children [17].

Since our model can effectively reflect the specific age-related methylation characteristics of children aged 0 to 18 years, it can be used to monitor abnormalities in children’s growth and development, as well as to predict the occurrence of diseases and the process of aging. Similar to bone age, which can be used to detect precocious puberty [39, 40], the DNA methylation age can be used to quantify the rate of aging in children. Our results revealed a pattern of changes in epigenetic age acceleration in healthy children. We attempted to explain this phenomenon by using the k-means clustering algorithm to analyze the variation in the β values of the 111 CpG sites (Supplementary Figure 5). The average β values of the sites from some clusters (e.g., cg26227465) were higher in toddlerhood, lower in mid-childhood and higher again in adolescence, whereas those from other clusters (e.g., cg25827666) were lower in toddlerhood, higher in mid-childhood and then slightly lower during adolescence. The cg26227465 site is located near the *IFNG* gene, which encodes a protein secreted by cells of both the innate and adaptive immune systems. This gene is associated with increased susceptibility to viral, bacterial and parasitic infections and to several autoimmune diseases [41–43]. The cg25827666 site is upstream of the *NTRK1* gene, which encodes a member of the neurotrophic tyrosine kinase receptor family. This kinase promotes cellular differentiation and may contribute to sensory neuron subtype specification [44, 45]. DNA methylation is considered an epigenetic marker of expression ability, as decreases in methylation are usually associated with increases in gene expression, and vice versa. Therefore, it was reasonable that the aging patterns of children were reflected in DNA methylation changes with increasing age.

Next, we applied our predictive model to children with autism and early lead exposure, and found that the DNA methylation age was accelerated in autistic children in mid-childhood and in boys exposed to lead. To demonstrate the legitimacy of our findings, we will now report our statistical power. First, the sample of 260 autistic children and 226 unaffected siblings was large enough to detect a significant AMAR difference of 0.1 between the two groups with adequate statistical power (91%). Previous studies have also provided some evidence of the accelerated aging of autistic patients. Autism is a lifelong condition [46] that increases the incidence of nearly all age-related health impairments in adulthood, including immune conditions, gastrointestinal and sleep disorders, seizures, obesity, dyslipidemia, hypertension and diabetes [47]. Autism can also markedly increase premature mortality [48] and reduce the quality of life [49]. Second, although the groups of 13 lead-exposed boys and 7 controls were relatively small, they also achieved 76% power to detect a significant AMAR difference of 0.4. Eid and Zawia also reported that lead induces brain aging and increases susceptibility to adult neurodegenerative diseases, particularly Alzheimer's disease and Parkinson's disease [50]. Given the improvements in our child-specific methylation-based age prediction model, we expect that it will be widely applied in research on pediatric health assessment and disease prevention. This could reveal aging trends with many practical implications.

Although our model can measure the biological ages of children more accurately than previous models, we do not currently have data on the outcomes of the included children later in life (e.g., risk of disease, time of death) to verify whether this new clock accurately measures biological aging caused by pediatric diseases. Therefore, Guangzhou Women and Children’s Medical Center is establishing pediatric disease cohorts for long-term follow-up. In this process, multi-omics data at different stages of life (continuing to adulthood and even to death) will be measured to test the ability of children’s biological clocks to characterize biological aging. Our pediatric cohort will also provide a larger database for this study, thus addressing the problem of the insufficient sample size and enabling us to explore the association of extensive disease outcomes with biological aging.

In conclusion, childhood (0–18 years) is the fastest period of development of various systems. Age-related DNA methylation changes in the peripheral blood of children occur more rapidly and with greater flexibility than those in adults. We established a methylation-based age prediction model specifically for children, which enabled us to quantify children’s biological ages with great accuracy, and to identify several determinants and variation trends of age acceleration in children. In addition to assessing the aging trends that correlated with epigenetic changes in childhood, we also investigated the effects of autism, GH deficiency and lead exposure on biological age in children. In future studies, our model can be used to identify other factors influencing the AAD and AMAR, including other childhood diseases or environmental factors (such as maternal smoking, alcohol intake or eating habits), and to quantify the impact of these factors on the health and longevity of children. Our biological age prediction model in children could be developed into a quantitative health assessment tool that detects health imbalances early in life, effectively preventing age-related diseases and postponing the aging process.

## MATERIALS AND METHODS

### Description of the datasets

We collected publicly available genome-wide methylation datasets of healthy children’s peripheral blood samples from the Gene Expression Omnibus database and other online resources to build our model. Details about the individual datasets (datasets 1–11) can be found in Table 1, along with the relevant citations. Dataset 1 consisted of leukocyte samples from 334 healthy (entirely male) subjects (mean age 10, range 3–17 years old) [17]. Dataset 2 included 13 unaffected subjects from a DNA methylation study of Crohn's disease and ulcerative colitis [51]. Dataset 3 comprised nine subjects of normal weight from an adolescent dietary fat study [52]. Dataset 4 involved 18 unaffected individuals from a study of age-related diseases [53]. Dataset 5 was obtained from a longitudinal analysis of genome-wide methylation changes in peripheral blood samples (n = 84) from healthy children before and after pubertal onset [18]. Dataset 6 consisted of 15 samples from a study on the effects of periconceptional maternal micronutrient supplementation on infant blood methylation patterns [54]. Dataset 7 was generated in the same lab as dataset 1, and contained samples from 127 healthy children measured on the Illumina 450K platform [17]. Dataset 8 included nine healthy boys and nine healthy girls who participated in a sex-specific DNA methylation analysis [55]. Dataset 9 comprised seven healthy control subjects from an analysis of co-methylation modules related to age [56]. Dataset 10 was obtained from the relatives of patients with Down Syndrome (mothers and unaffected siblings) [57]. Dataset 11 included 88 lean individuals aged 14 to 16 years who were recruited by mail and through school visits in Uppsala, Sweden [58]. Five datasets were obtained from Illumina 27K arrays, while six were obtained from Illumina 450K arrays.

The datasets of children with diseases are summarized in Supplementary Table 1 (datasets 1–3). These datasets were also obtained from the Gene Expression Omnibus database. Dataset 1 contained methylation data on 27,578 CpG dinucleotides in peripheral blood leukocyte DNA samples from autistic children and unaffected siblings [17]. Dataset 2 included samples from 24 patients at baseline and after four days of recombinant human growth hormone (rhGH) treatment [27]. Dataset 3 consisted of 42 dry blood spots from children exposed to lead [30].

### DNA methylation data pre-processing and quality control

The public Illumina DNA data described above were generated with either the Illumina Infinium HumanMethylation27 BeadChip or the Illumina Infinium HumanMethylation450 BeadChip. Both arrays are used to quantify DNA methylation based on β values, which range from 0 (completely unmethylated) to 1 (completely methylated). We merged the data from the two platforms by focusing on the ~26,000 CpG sites that are present in both platforms. The age prediction model was trained on 21,979 probes that were shared between the Illumina 27K and 450K platforms and had ≤ 10 missing values across the datasets. Then, the R ‘impute’ package was used to impute the remaining missing values with the k-nearest-neighbors approach (10 nearest markers) [59]. The BMIQ R function [60] was used to readjust the 21,000 overlapping probes so that their distribution met the gold standard (the mean β value of the largest single dataset (GSE27097) in this article [17]).

We performed a principal component analysis to identify and remove outliers. First, each sample was converted into a z-score statistic based on the squared distance of the first principal component from the population mean. Then, the z-score was converted to the false-discovery rate through the Gaussian cumulative distribution function and the Benjamini-Hochberg procedure [61]. Samples falling below a false-discovery rate of 0.2 were designated as outliers and were removed. This filtering procedure was performed iteratively until no samples were determined to be outliers. The remaining 716 samples were used in the age prediction model. Specific information on these samples is shown in Table 1.

### Age conversion and DNA methylation age prediction model

To improve the accuracy of the prediction model, we used months as the age unit. In the included datasets, 84% of the sample ages were recorded as months. The sample ages recorded as years were converted to months for this study. We employed k-fold cross-validation (k = 10) in the R ‘caret’ package [22] to randomly cleave the datasets 10 times and build a model for each cohort. During each run, a different cluster was used as the test set, and the remaining clusters were used as the training set, with proportions of 10% and 90%, respectively.

Based on the training set data, we found it advantageous to transform age using function F before building the prediction model. Using the inverse of function F, we transformed the linear part of the regression model into the DNA methylation age. Function F was as follows (toddler.age was set to 48 months):

$$\begin{array}{l} F\left(age\right)=log\left(age+1\right)\\ -log\left(toddler.age+1\right)ifage\leq toddler.age \end{array}$$

$$\begin{array}{l} F\left(age\right)\\ =\left(age-toddler.age\right)/\left(toddler.age+1\right)ifage\\ >toddler.age \end{array}$$

The child-specific biological age prediction1 model was established through sure independence screening combined with multivariate linear modeling based on the elastic net algorithm. First, we used sure independence screening (implemented in the R package ‘SIS’) [20] to reduce the dimensionality of the ~21,000 β values in the datasets. This step was taken because variable selection methods (e.g., lasso, LARS, SCAD) do not perform well when the dimension of the predictor variable p is much larger than the sample size n. Then, an elastic net regression model (implemented in the glmnet R function) [21] was used to regress a transformed model of age based on 111 β values in the training data. The elastic net approach is a combination of traditional lasso and ridge regression methods, emphasizing model sparsity while appropriately balancing the contributions of correlated variables. The glmnet function requires the user to specify two parameters (alpha and lambda). Since we used an elastic net predictor, alpha was set to 0.48, and lambda was set to 0.000954 based on 10-fold cross-validation of the training data (via the R function cv.glmnet). A heat map was drawn in the ‘pheatmap’ package in RStudio, and Venn diagrams were produced on the Bioinformatics and Evolutionary Genomics website (http://bioinformatics.psb.ugent.be/webtools/Venn/).

### CpG site annotation and enrichment analysis

The Entrez gene IDs of CpG sites in the HumanMethylation27 and HumanMethylation450 annotation files were used to identify genes. Gene Ontology (GO) and Kyoto Encyclopedia of Genes and Genomes (KEGG) analyses were conducted in R with the ‘clusterProfiler’ package from Bioconductor [62]. Enrichment analyses were performed with Fisher’s exact test. Significant GO terms (*P* < 0.05) were imported into REVIGO for visualization in a semantic space [63].

### Statistical analysis

Paired Student’s t-tests were used to compare the DNA methylation ages calculated by our model and the multi-tissue predictor for 67 pairs of monozygotic twins in the dataset GSE56105 [23]. Differences between two groups of samples were assessed with Wilcoxon tests and unpaired Student’s t-test, while differences among multiple groups of samples were assessed with analysis of variance (ANOVA). *P* values < 0.05 were considered significant. Statistical analyses were performed with RStudio.

## Supplementary Material

Supplementary Figures

Supplementary Tables

Supplementary Table 2

Supplementary Table 3

Supplementary Table 4
