# Supplementary material for: DNA methylation profile is a quantitative measure of biological aging in children
Source: Aging (Albany NY). 2019 Nov 22;11(22):10031–51. doi: 10.18632/aging.102399 (PMC6914436; doi:10.18632/aging.102399)
Supplement: Supplementary Figures [file aging-11-102399-s005..pdf]

## SUPPLEMENTARY FIGURES

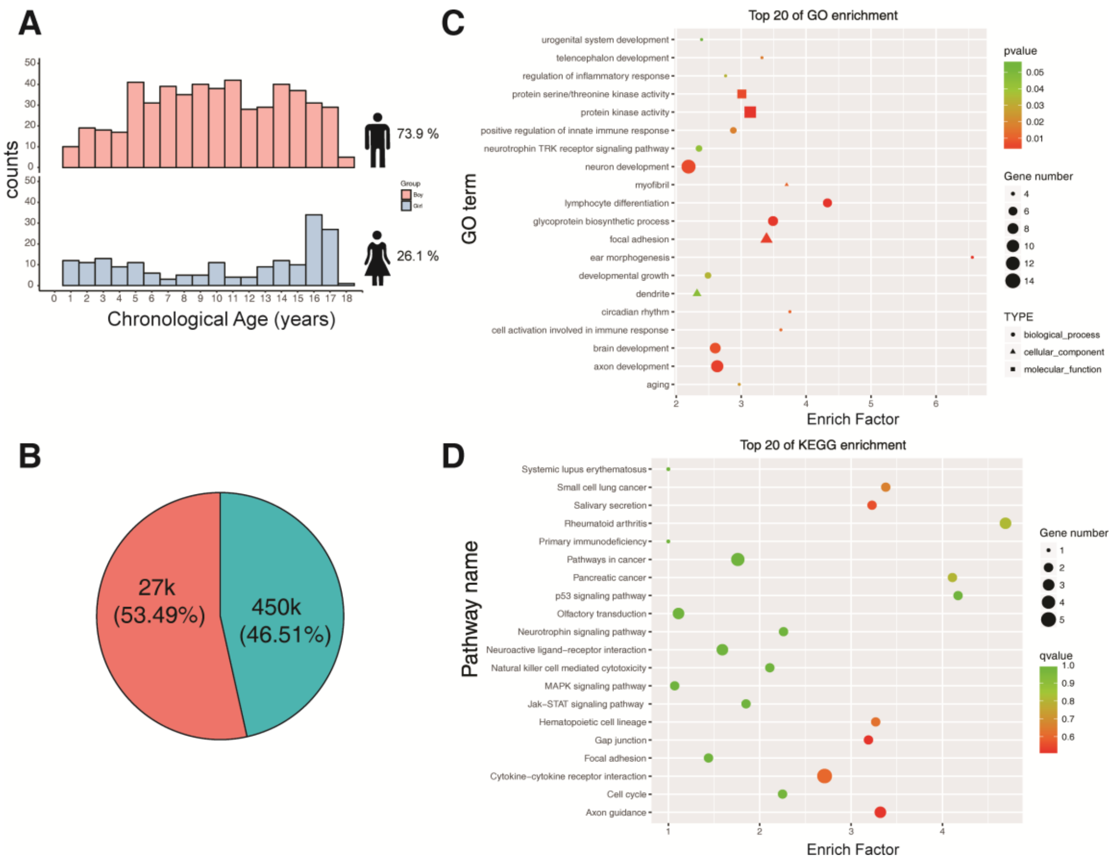


**Supplementary Figure 1. Characteristics of the prediction model.** (**A**) Histograms of the age distribution for healthy girls and boys, respectively. The *x*-axis represents the chronological age of the individuals (age unit is years) and the *y*-axis (counts) represents the number of individuals. The pink columns indicate boys, and the blue columns indicate girls. (**B**) Pie chart displaying the proportion of the different types of Illumina Methylation Assays. Red stands for the 27K assay and green stands for the 450K assay. (**C**) Gene Ontology (GO) analysis of the genes associated with the 111 CpG sites. (**D**) Kyoto Encyclopedia of Genes and Genomes (KEGG) pathway analysis of the genes associated with the 111 CpG sites.

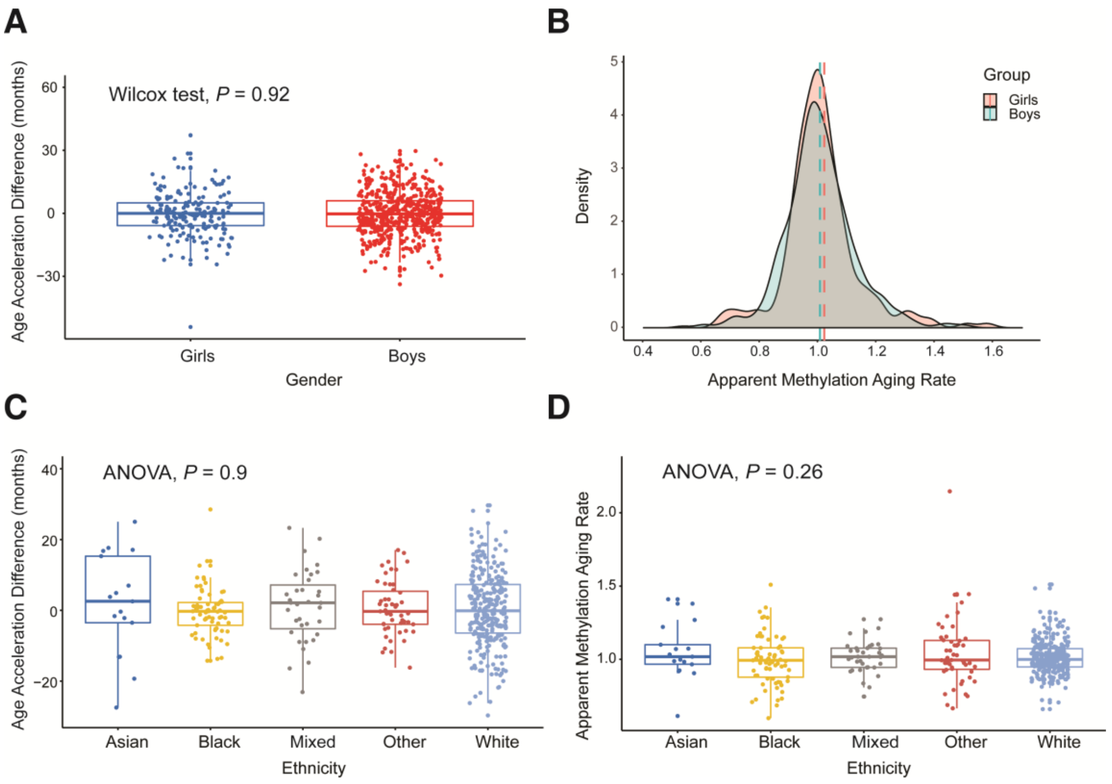


**Supplementary Figure 2. Age acceleration in children grouped by gender and ethnicity.** (**A**) Boxplot comparing the age acceleration difference between girls and boys. (**B**) Density plot of the apparent methylation aging rates of children separated by gender. Dashed lines represent the mean values. (**C**) Boxplot comparing the age acceleration differences among children of different ethnicities. (**D**) Boxplot comparing the apparent methylation aging rates among children of different ethnicities.

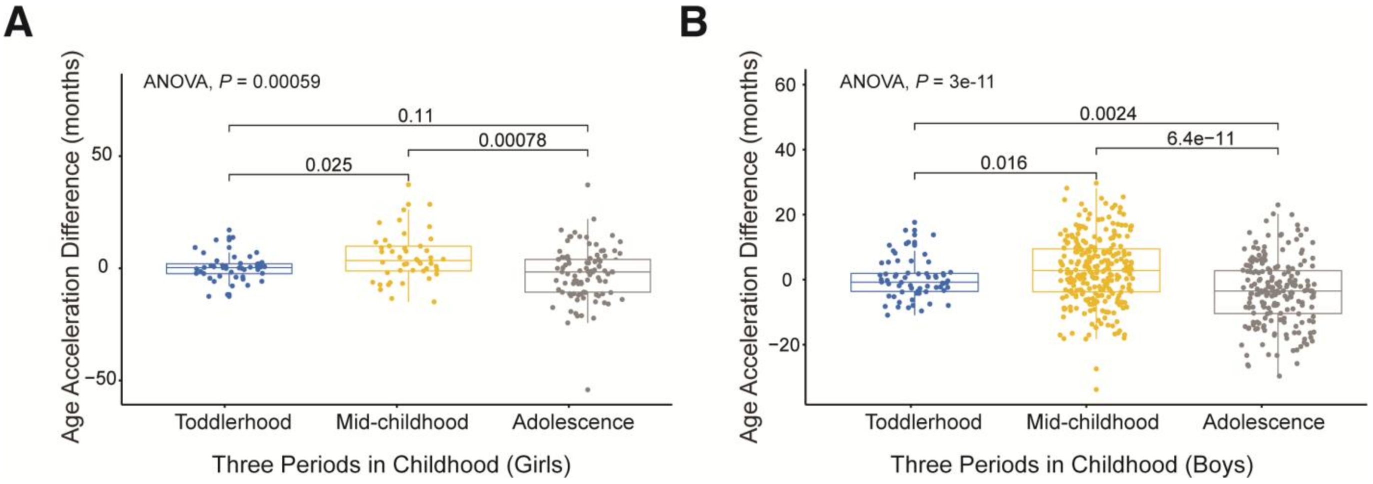


**Supplementary Figure 3. Age acceleration in different periods of childhood by gender.** (**A**) Boxplot of the age acceleration difference during different periods of childhood in girls. (**B**) Boxplot of the age acceleration difference during different periods of childhood in boys.

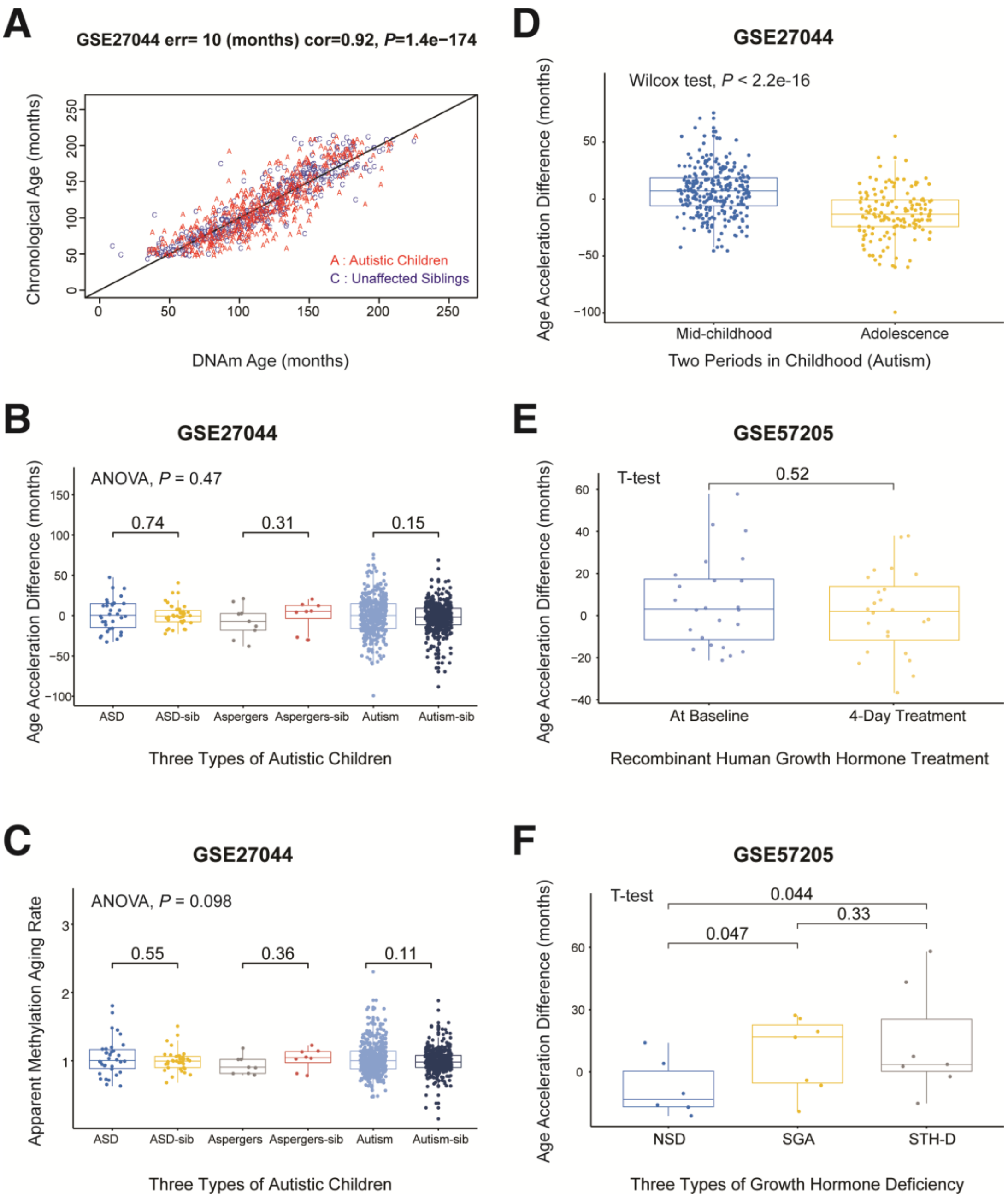


**Supplementary Figure 4. Age acceleration in children with diseases.** (**A**) Scatterplot of the DNA methylation (DNAm) age (*x*-axis) against the chronological age (*y*-axis) in the GSE27044 dataset (age unit is months). In this dataset, the correlation between the DNAm age and the chronological age was 0.92, and the error (median absolute difference) was 10 months. ‘A’ stands for autistic children and ‘C’ stands for their unaffected siblings. (**B**) Boxplot comparing the age acceleration differences among children with different types of autism and their unaffected siblings (‘-sib’). (**C**) Boxplot comparing the apparent methylation aging rates among children with different types of autism. (**D**) Boxplot comparing the age acceleration differences in mid-childhood and adolescence in children with autism. (**E**) Boxplot comparing the age acceleration differences before and after four days of recombinant human growth hormone treatment. (**F**) Boxplot comparing the age acceleration differences among children with different types of growth hormone deficiency.

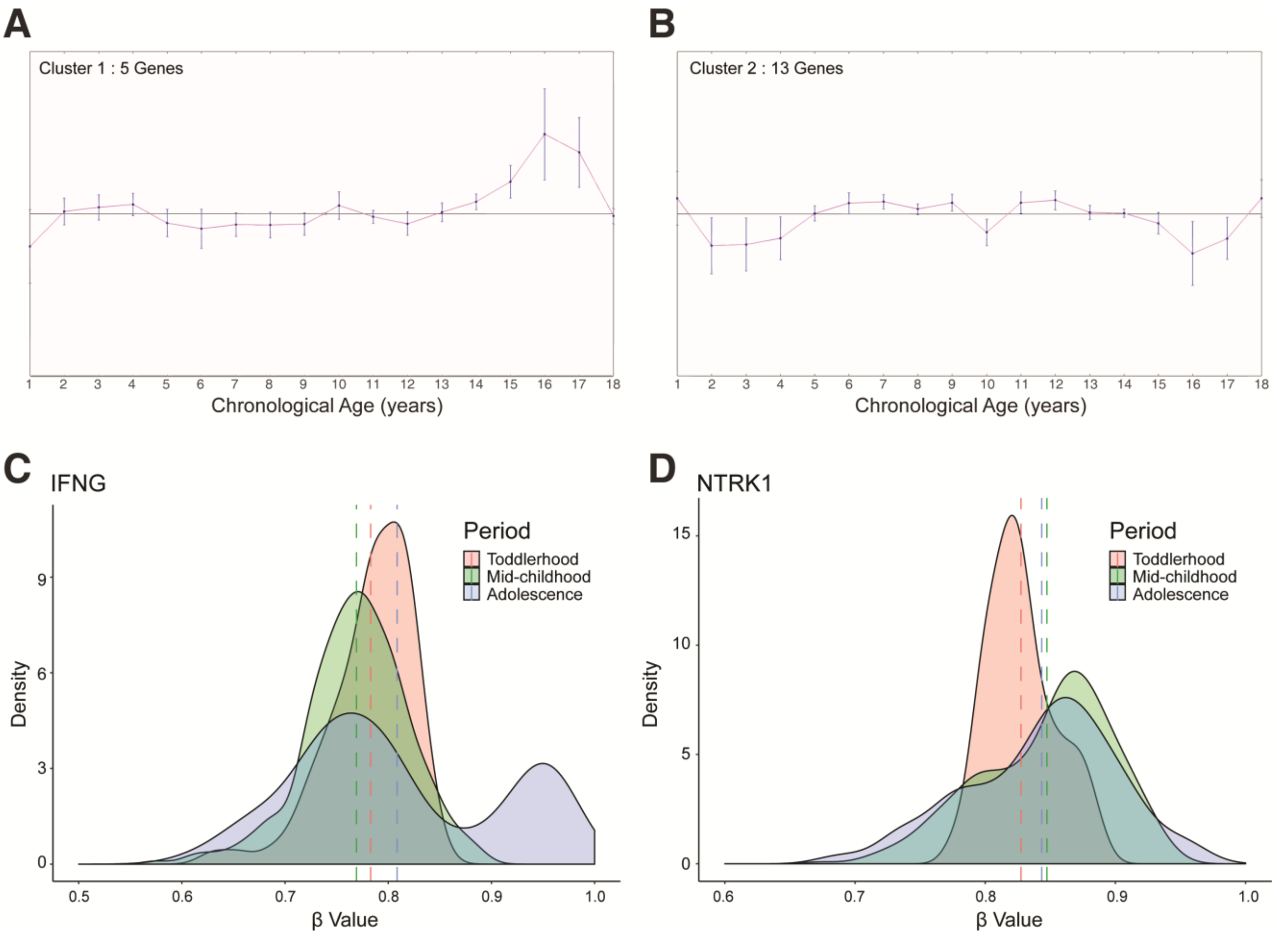


**Supplementary Figure 5. K-means clustering analysis of the 111 CpG sites.** (**A**) The trend of DNA methylation levels and the number of genes associated with the CpG sites in Cluster 1. (**B**) The trend of DNA methylation levels and the number of genes associated with the CpG sites in Cluster 2. (**C**) Density plot of the β value of cg26227465, located near the *IFNG* gene, during different periods of childhood. This site was in Cluster 1. (**D**) Density plot of the β value of cg25827666, located near the *NTRK1* gene, during different periods of childhood. This site was in Cluster 2.
