# Supplementary material for: DNA methylation profile is a quantitative measure of biological aging in children
Source: Aging (Albany NY). 2019 Nov 22;11(22):10031–51. doi: 10.18632/aging.102399 (PMC6914436; doi:10.18632/aging.102399)
Supplement: Supplementary Tables [file aging-11-102399-s004..pdf]

SUPPLEMENTARY TABLES

Supplementary Table 1. Summary details of the DNA methylation datasets on children in disease.

ID	Availability	Methylation array	n	Age(months)	Gender	Disease	Ethnicity	Citation
1	GSE27044	Illumina 27K	866	43.0-214.0	M: 866	Autism: 397; Autism-sib: 382; ASD: 34; ASD-sib: 36; Aspergers: 9; Aspergers-sib: 8;	White: 698; Asian: 32; African-amer: 14; other: 25; more-than-one-race: 80; Native-american: 2; Native-hawaiian: 2; not-specified: 13;	Alisch et al.
2	GSE57205	Illumina 450K	48(24)	62.0-185.0	M: 14 F: 10	NSD: 6; SGA: 7; STH-D: 7; Q-STH-D: 2; IGF1-D: 1; UTS: 1;	null	Kolarova et al.
3	GSE60598	Illumina 27K	42	3.0-60.0	M: 24 F: 18	BLL ≥ 5 μg/dl: 25; BLL < 5 μg/dl: 17;	African_american: 41; Caucasion_mixed: 1	Sen et al.

1. NSD: GH deficiency due to neurosecretory dysfunction; SGA: small for gestational age; STH-D: classical GH deficiency; Q-STH-D: qualitative GH deficiency (Kowarski syndrome); IGF1-D: IGF1 deficiency; UTS: Turner-Syndrome;
2. GSE57205 contains 48 blood samples from 24 patients: 24 samples at baseline and 24 samples after 4-day short-term recombinant human growth hormone treatment stimulation;
3. BLL: blood lead level.

Please browse Full Text version to see the data of Supplementary Tables 2 to 4.

Supplementary Table 2. The regression coefficients of child-specific methylation-based age prediction model.

Supplementary Table 3. The result of REVIGO on the significant GO terms.

Supplementary Table 4. The predict details of GSE27044 dataset.
